# PLGA Nanoparticles Double-Decorated with a TAT Peptide and Folic Acid to Target *Staphylococcus aureus*

**DOI:** 10.3390/ijms262110666

**Published:** 2025-11-01

**Authors:** Stéphanie Andrade, Maria J. Ramalho, João Santos, Sílvio Santos, Luís D. R. Melo, Nuno Guimarães, Maria P. Ferraz, Nuno F. Azevedo, Maria C. Pereira, Joana A. Loureiro

**Affiliations:** 1 LEPABE, ALiCE, Faculty of Engineering, University of Porto, Rua Dr. Roberto Frias, 4200-465 Porto, Portugal; stephanie@fe.up.pt (S.A.); mjramalho@fe.up.pt (M.J.R.); up202103293@edu.fe.up.pt (J.S.);; 2 CEB—Centre of Biological Engineering, University of Minho, 4710-057 Braga, Portugal; silviosantos@ceb.uminho.pt (S.S.);; 3 LABBELS—Associate Laboratory, University of Minho, 4710-057 Braga, Portugal; 4 Faculty of Pharmacy, University of Coimbra, 3000-548 Coimbra, Portugal; 5 Department of Mechanical Engineering, Faculty of Engineering, University of Porto, 4200-465 Porto, Portugal; mpferraz@fe.up.pt; 6 i3S—Institute for Research and Innovation in Health, University of Porto, 4200-135 Porto, Portugal; 7 INEB—Institute of Biomedical Engineering, University of Porto, 4200-135 Porto, Portugal

**Keywords:** cell-penetrating peptide, drug delivery system, antimicrobial resistance, bacteria targeting, biocompatibility

## Abstract

Treating bacterial infections has become increasingly difficult due to the rise in antibiotic-resistant bacterial strains. Strategies involving the targeted delivery of antibiotics have been proposed to minimize the administered antibiotic doses. This study aims to develop the first double-modified nanovehicle capable of increasing bacterial membranes’ permeability while specifically targeting *Staphylococcus aureus*, one of the foremost pathogens responsible for global mortality rates. Thus, polymeric NPs composed of poly(lactic-co-glycolic acid) (PLGA) were produced, and their surface was modified with TAT peptide to increase the membranes’ permeability and folic acid (FA) to direct the NPs to *S. aureus*. The nanosystem showed spherical morphology with sizes of 174 ± 4 nm, a monodisperse population (polydispersity index of 0.08 ± 0.02), and a zeta potential of −2.5 ± 0.1 mV. The NPs remained stable for up to four months during storage. Fluorescence-based flow cytometry analysis proved that the double modification of PLGA NPs increased the interaction of the NPs with *S. aureus*, with fluorescence increasing from 71 ± 3% to 87 ± 1%. The nanosystem slightly affected the growth curve of *S. aureus* by extending both the lag time (from 2.5 ± 0.2 to 2.88 ± 0.4 h) and the exponential phase, as evidenced by an increase in the half-maximum growth time (from 3.9 ± 0.2 to 4.4 ± 0.1 h). Furthermore, the nanocarrier showed no toxicity for human dermal fibroblast cells, maintaining a 100% cell viability at the highest concentration tested (100 µM). Therefore, the proposed FA/TAT-functionalized nanocarrier presented promising features to be successfully used as a delivery vehicle of antimicrobials to fight *S. aureus*.

## 1. Introduction

The world is grappling with a global health emergency characterized by severe illness and high mortality rates caused by infectious diseases. According to Ikuta et al. [1], bacterial infections were identified as the cause of 7.7 million deaths worldwide, representing almost 14% of total global mortality. This places bacterial infections as the second leading cause of death globally, following ischemic heart disease.

Among the approximately 1400 human pathogens that have been described so far [2], *Staphylococcus aureus* (*S. aureus*) is the leading pathogen causing death globally, being the only organism causing more than 1 million deaths in 2019 [1,3]. These numbers are highly connected to clinically relevant resistance mechanisms of *S. aureus* against available antibiotics. Such resistance, which arose from the frequent and suboptimal use of antibiotics to treat *S. aureus*-induced infections, significantly amplifies the likelihood of antibiotic treatment failure [4].

Despite the continued emergence and spread of antibiotic-resistant *S. aureus*, antibiotics remain the primary treatment for *S. aureus* infections. However, the current state of antibiotic development is stagnant, with few new antibiotics reaching the market. Hence, there is a critical need to develop innovative strategies for the effective delivery of existing antibiotics capable of overcoming the resistance mechanisms of *S. aureus* and, consequently, enhancing antibiotics’ efficacy [5]. Utilizing nanoparticles (NPs) as drug delivery systems (DDSs) has emerged as an effective approach to overcome bacterial resistance. NPs have been shown to protect loaded antimicrobials from biological degradation and efflux pumps [6]. Additionally, NPs enable controlled and sustained drug release, ensuring the maintenance of active therapeutic levels over prolonged periods [7].

Despite their therapeutic effectiveness compared to conventional dosage forms, achieving precise targeting of NPs to the infection site is essential for effectively treating bacterial infections. This targeted approach ensures the delivery of concentrated antimicrobials directly to the site of infection. Consequently, lower doses of antimicrobials are required to achieve therapeutic effects, thus minimizing potential drug side effects on healthy cells and tissues [8].

Recently, various drug-loaded NPs have been reported for the targeted treatment of *S. aureus* infections. These include gold NPs [9], polymeric NPs [10], liposomes [11], solid lipid NPs [12], lipid-polymer hybrid NPs [13], and mesoporous silica NPs [14]. However, none of them has achieved significant progress towards clinical approval, which may be related to the difficulty of NPs in crossing biological and bacterial membranes. Thus, this study aims to pioneer the development of the first double-decorated nanocarrier, with the unique capability of enhancing membranes’ permeability while specifically targeting *S. aureus*. For that, polymeric NPs made of poly(lactic-co-glycolic acid) (PLGA) were prepared as DDS due to their biodegradability, biocompatibility, non-immunogenicity, high stability in body fluids, and FDA-approved use. These NPs can encapsulate hydrophilic and hydrophobic compounds, making them suitable for a wide range of therapeutic agents [15]. PLGA NPs’ surface was then modified with a cell-penetrating peptide (TAT) to enhance NPs’ transportation across membranes [16] and with folic acid (FA), to facilitate the targeted delivery of the NPs to *S. aureus*. This modification was based on the overexpression of folate receptors in *S. aureus*-infected tissues compared to healthy tissues [17]. This innovative nanovehicle was thoroughly characterized, and its in vitro bacterial targeting ability and antibacterial properties towards *S. aureus* were investigated. The in vitro NPs’ biocompatibility was also examined in human dermal fibroblast cells (HDFn).

## 2. Results

### 2.1. Physicochemical Characterization of NPs

**Figure 1:**
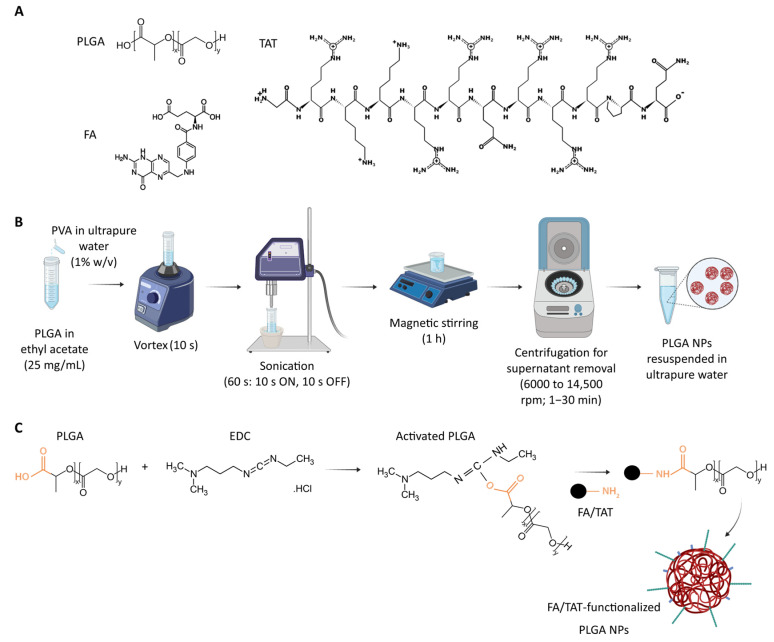
(**A**) Chemical structure of poly(lactic-co-glycolic acid) PLGA, folic acid (FA), and TAT. (**B**) Schematic illustration of PLGA nanoparticles (NPs) preparation by the single emulsion-solvent evaporation technique and (**C**) their functionalization with FA and TAT by 1-ethyl-3-(3-dimethylaminopropyl)carbodiimide (EDC) coupling reaction.

In this work, PLGA NPs were prepared using the single emulsion-solvent evaporation technique. The NPs’ surface was then functionalized with FA, TAT, or FA/TAT using the water-soluble carbodiimide coupling reagent *N*-(3-dimethylaminopropyl)-N′-ethylcarbodiimide hydrochloride (EDC). Figure 1 displays the chemical structure of PLGA, FA, and TAT (Figure 1A) and a schematic illustration of the PLGA NPs’ preparation (Figure 1B) and functionalization (Figure 1C).

Table 1 shows the physicochemical properties of the prepared non-functionalized and FA-, TAT-, and FA/TAT-functionalized PLGA NPs in terms of hydrodynamic diameter, polydispersity index (PDI), and zeta potential.

The size of NPs plays a critical role in multiple aspects, including their ability to cross biological barriers like the blood–brain barrier, maintain stability within the body, circulate in the bloodstream, release drugs effectively, be taken up by cells, be cleared from the body, and avoid causing toxicity. As shown in Table 1, non-functionalized PLGA NPs presented a mean hydrodynamic diameter of 159 ± 12 nm. A significant increase in the mean diameter of the NPs was observed after their functionalization with FA, TAT, or FA/TAT (*p* < 0.05), which suggests the successful functionalization of the molecules at the NPs’ surface. These results align with other works, which also observed an increase in PLGA NPs’ size after FA [18,19] and TAT conjugation [20,21]. Additionally, no significant differences (*p* > 0.05) were observed between the size of FA-, TAT-, or FA/TAT-functionalized NPs. According to recent evidence, NPs larger than 200 nm have been demonstrated to activate the lymphatic system and leave the circulation more quickly than smaller NPs [22]. Moreover, NPs smaller than 100 nm can easily cross biological membranes. However, their higher surface-to-volume ratio makes them more toxic than larger NPs of the same composition. Thus, NPs’ ideal diameter has been defined to be 100 to 200 nm [23]. Therefore, regardless of the NPs’ modification, all formulations showed appropriate sizes to be used as DDS.

In addition to suitable mean sizes, all formulations presented PDI values lower than 0.08, with no significant differences between groups (*p* > 0.05) (Table 1). These values indicate highly monodisperse populations of NPs with a narrow size distribution. PDI values lower than 0.2 are generally accepted for drug delivery purposes [24]. The intensity-based size distribution curves obtained by dynamic light scattering (DLS) are provided in Figure S1 (Supplementary Information). As observed, all samples exhibited monomodal and symmetric distributions with narrow peaks, confirming the homogeneity of the NPs and the absence of aggregation after functionalization.

Zeta potential is a measure of the NP’s surface charge and, thus, another key parameter regulating the biodistribution, pharmacokinetic properties, and toxicity of the NPs in vivo [25]. Typically, PLGA NPs present a negative surface charge due to the negative charge of PLGA’s carboxyl group [26]. However, as revealed by Table 1, PLGA NPs in HEPES buffer displayed a zeta potential of −3.6 ± 1.7 mV. The ionic concentration of the dispersant is an important factor influencing the composition of the diffuse layer and, consequently, the NPs’ zeta potential [27]. For instance, Inam et al. [28] observed a reduction in the zeta potential of PLGA NPs (absolute value) with increasing HEPES buffer concentration. It is predictable that the two nitrogen atoms of HEPES interact with PLGA’s carboxyl groups, thus masking the PLGA NPs’ negative charge [28]. For the same reason, no significant alterations in the NPs’ zeta potential after FA, TAT, or FA/TAT modification were observed (*p* > 0.05), despite the charge of FA (−2) and TAT (+8) at physiological pH [29]. Therefore, the zeta potential of non-functionalized and FA-, TAT-, and FA/TAT-functionalized PLGA NPs was revealed to be suitable for drug delivery applications.

**Figure 2:**
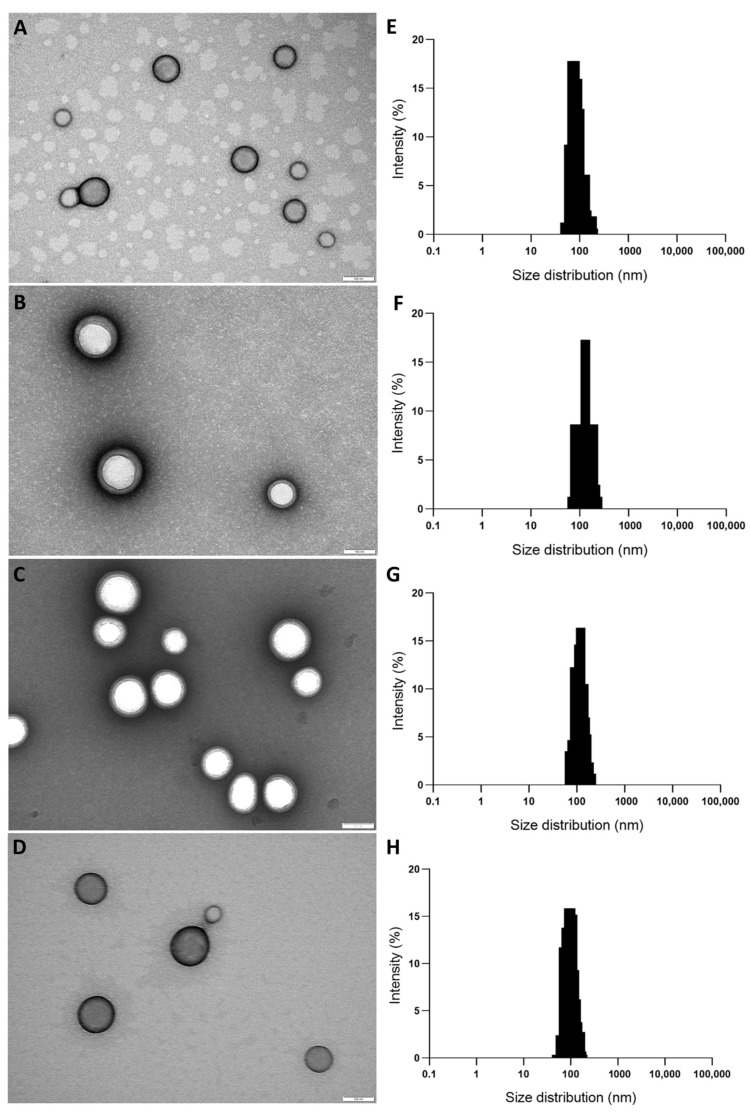
Transmission electron microscopy **(**TEM) images of (**A**) non-decorated, and (**B**) FA-, (**C**) TAT-, (**D**) FA/TAT-decorated PLGA NPs. Images obtained at 100,000× magnification. Scale bars represent 100 nm. Size distribution histograms of (**E**) non-decorated, (**F**) FA-, (**G**) TAT-, (**H**) FA/TAT-decorated PLGA NPs. Scales bars correspond to 100 nm.

The morphology of non-decorated and FA-, TAT-, and FA/TAT-decorated PLGA NPs was then examined by transmission electron microscopy (TEM). As shown in Figure 2A–D, TEM images of all formulations showed uniform NPs with well-defined spherical shapes. Furthermore, Figure 2E–H show the size distribution histograms of non-decorated and FA-, TAT-, and FA/TAT-decorated PLGA NPs, respectively. The figures display a monodisperse population of NPs, with the absence of aggregates, which is confirmed by the PDI below 0.1.

Several reports indicate that the geometry of NPs significantly influences their interaction with cells, with spherical particles often demonstrating superior uptake compared to rod-shaped or elliptical counterparts [30]. However, this trend is not absolute and can vary depending on both the specific cell type and the size range of the nanoparticles. In the present study, the particles observed under microscopy appeared slightly smaller than the sizes recorded through DLS. This variation is primarily due to the principles underlying DLS measurements, which capture the hydrodynamic diameter rather than the true physical dimensions. The hydrodynamic size incorporates contributions from factors such as the surrounding solvent layer, particle–particle interactions, and size distribution heterogeneity, which together can result in measurements that overestimate the actual nanoparticle size [31]. In turn, this trend has also been associated with the vacuum environment of TEM, which can lead to the dehydration and subsequent collapse of the hydrated polymer layer on the NPs’ surface, resulting in smaller sizes compared to DLS measurements [32]. Understanding this distinction is essential for accurately interpreting size-dependent behaviors, including cellular uptake, circulation dynamics, and overall biological performance of the nanoparticles.

**Figure 3:**
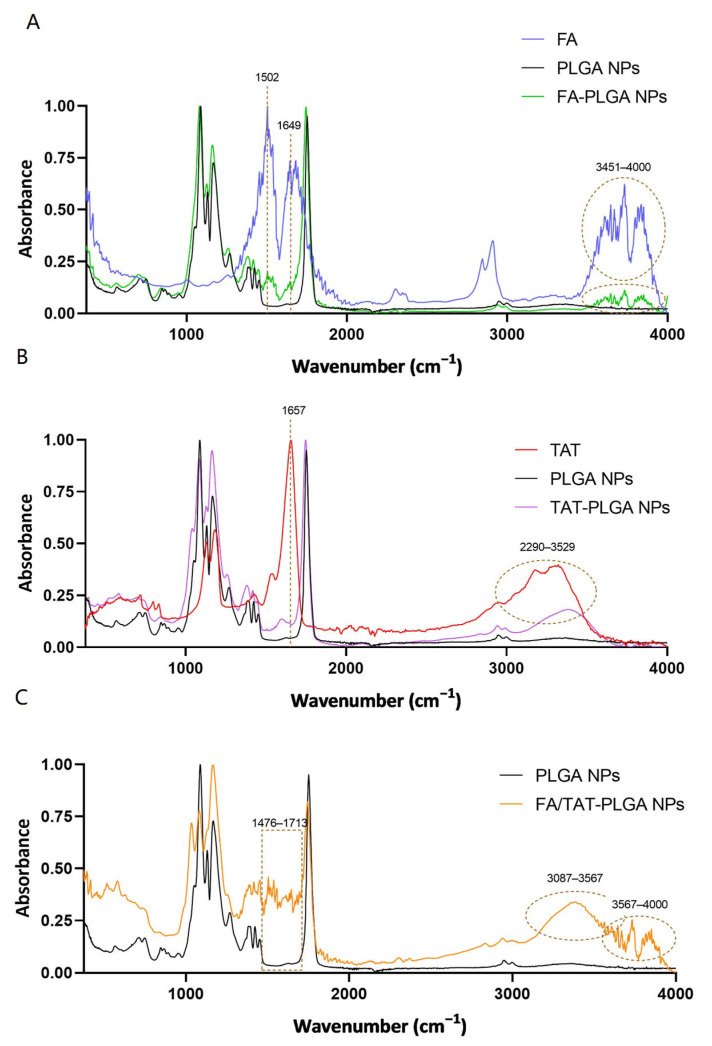
Fourier transform infrared spectroscopy (FTIR) spectra of (**A**) FA (blue), non-decorated (black), and FA-decorated PLGA NPs (green), (**B**) TAT (red), non-decorated (black), and TAT-decorated PLGA NPs (purple), and (**C**) non-decorated (black) and FA/TAT-decorated PLGA NPs (orange).

The success of PLGA NPs’ modification was confirmed by Fourier-transform infrared spectroscopy (FTIR) analysis. FTIR spectra of PLGA NPs before and after surface modification with FA, TAT, and FA/TAT are shown in Figure 3. Spectra of FA and TAT are also provided for comparison.

As depicted by the black lines (Figure 3), the FTIR spectrum of PLGA NPs is characterized by a peak at 1752 cm^−1^ and a band between 1026 and 1223 cm^−1^, attributed to the stretch of C=O and C-O, respectively [33]. Concerning FA (Figure 3A, blue line), two major peaks at 1502 and 1649 cm^−1^ are observable, ascribed to the C-C stretch of the phenyl ring and the N-H bend of the pteridine ring, respectively. Moreover, a band between 3451 and 4000 cm^−1^ is noticeable, which corresponds to the O-H stretch of the glutamic acid moiety and N-H of the pteridine ring [34]. The presence of the mentioned bands in the spectrum of FA-decorated PLGA NPs (Figure 3A, green line) confirms the conjugation of FA to the NPs.

In turn, the TAT peptide (Figure 3B, red line) exhibited characteristic bands corresponding to the vibration of the amide I and II bonds at 1657 and 1537 cm^−1^, respectively. These vibrations are, respectively, linked to the C=O stretching and N-H bending of the amide bonds between TAT’s amino acids [35]. A band between 1055 and 1216 cm^−1^ was also identified as corresponding to C–-O ester stretch vibrations from the C-terminal amino acid of TAT [36]. Additionally, a broad band between 2290 and 3529 cm^−1^ is observed, corresponding to N-H stretching vibrations of TAT’s amino groups [35]. Identical bands are observed in the FTIR spectrum of TAT-decorated NPs, validating the modification of PLGA NPs with TAT.

Lastly, it is possible to verify from the orange line of Figure 3C that the aforementioned characteristic bands of FA and TAT are also present in the FTIR spectrum of FA/TAT-PLGA NPs. In fact, a broad band between 1476 and 1713 cm^−1^, corresponding to the C-C and C=O stretches as well as the N-H bonds of both molecules, is visible. Moreover, the orange spectrum shows bands at 3087–3567 and 3567–4000 cm^−1^, which are bands identified in the FTIR spectra of TAT and FA, respectively.

To confirm that the changes observed in the FTIR spectra of PLGA NPs resulted from the chemical conjugation of FA and/or TAT rather than simple physical mixing, FTIR spectra of physical mixtures of PLGA NPs with FA, TAT, or FA/TAT were also recorded, as shown in Figure S2 (Supplementary Information). The resulting spectra were similar to those of non-decorated PLGA NPs, with no additional peaks observed. This can be explained by the fact that, in the physical mixtures, the relative amount of FA or TAT is very small, and their characteristic vibrational bands are largely masked by the dominant PLGA signal. In contrast, during chemical conjugation, the amino groups of FA or TAT react with the carboxylic groups of PLGA, leading to the formation of additional amide bonds. These bonds give rise to new characteristic peaks clearly visible in the spectra of the functionalized NPs (Figure 3). Therefore, FTIR analysis confirms the effective modification of the NPs with both FA and TAT.

### 2.2. Physicochemical Stability of NPs

**Figure 4:**
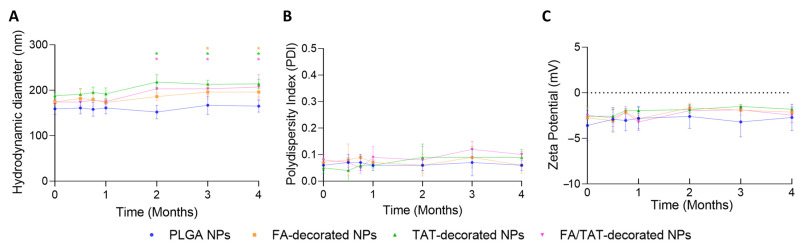
(**A**) Hydrodynamic diameter, (**B**) polydispersity index (PDI), and (**C**) zeta potential of non-decorated (blue) and FA-(orange), TAT-(green), and FA/TAT-decorated PLGA NPs (pink) in HEPES buffer over 4 months. Samples were stored at 4 °C during the stability study. * Indicates a statistical difference compared to the PLGA NPs at the beginning of the experiment (*p* < 0.05).

One major barrier to NPs’ commercialization is still their physicochemical stability. Because of this, it is essential to confirm that NPs’ physicochemical characteristics are maintained throughout the storage term in order to guarantee their stability. Changes in the NPs’ physicochemical properties, such as mean size, PDI, and zeta potential, are indicators of instability. Therefore, the physicochemical stability of non-decorated and FA-, TAT-, and FA/TAT-decorated PLGA NPs at storage temperature (4 °C) was investigated over 4 months by observing their physical appearance and measuring their mean size, PDI, and zeta potential periodically (Figure 4).

Results demonstrate that all the evaluated properties for non-decorated PLGA NPs did not change significantly over 4 months at 4 °C (*p* > 0.05), implying that the NPs remained stable during this period. Moreover, the hydrodynamic diameter, PDI, and zeta potential of PLGA NPs functionalized with FA, TAT, or both molecules also remained constant (*p* > 0.05) after 1 month under storage conditions. However, after 2 months, a slight increase in the mean size of TAT and FA/TAT-modified NPs, corresponding to 12 and 17%, respectively, was verified (*p* < 0.05), which remained unchanged until the end of the experiment (*p* > 0.05). Concerning FA-modified NPs’ diameter, an increase of 10% was observed after 4 months of storage (*p* < 0.05). However, no variations in zeta potential and PDI values were observed over the duration of the stability study (*p* > 0.05), inferring that modified NPs maintained their narrow size distribution. Additionally, no visible indications of phase separation, flocculation, or creaming were detected. Overall, the results indicate the suitable colloidal stability of non-decorated and FA-, TAT-, and FA/TAT-decorated PLGA NPs at storage temperature.

### 2.3. Bacterial Targeting Ability of NPs

To explore the targeting ability of the double-modified nanocarrier, PLGA NPs with or without the surface’s double modification were loaded with the fluorescent molecule rhodamine B and incubated with *S. aureus* at 37 °C for 1 h to allow NP–bacteria interactions. No significant changes in particle size, PDI, or zeta potential were observed after dye encapsulation, as detailed in Table S1 (Supplementary Information), confirming that the loading process did not affect the NPs’ physicochemical properties.

**Figure 5:**
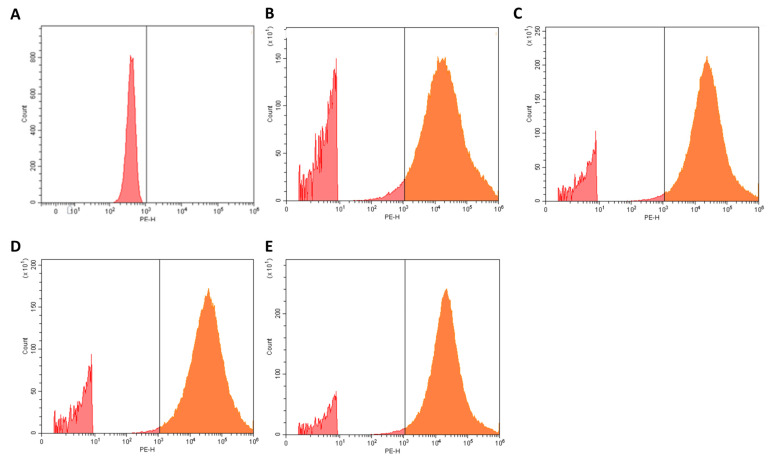
Representative images of flow cytometry results of *S. aureus* (orange data) (**A**) without treatment or treated with (**B**) non-modified rhodamine B-loaded PLGA NPs or (**C**) rhodamine B-loaded FA-modified PLGA NPs, (**D**) rhodamine B-loaded TAT-modified PLGA NPs, or (**E**) rhodamine B-loaded FA/TAT-modified PLGA NPs. The peaks to the left of the vertical central lines (red data) are related to autofluorescence of bacteria.

Importantly, HEPES buffer was used in this study to closely mimic physiological conditions, including pH and ionic strength. While HEPES may partially influence the ionization state of TAT, potentially modulating its cell-permeating ability, HEPES buffer was chosen to ensure that the observed interactions reflect a biologically relevant environment. Fluorescence-based flow cytometry analysis was conducted, and representative images of the results are displayed in Figure 5.

As shown in Figure 5A, *S. aureus* without treatment with NPs (control) shows no fluorescence, as expected [37]. On the contrary, after incubation of *S. aureus* with rhodamine B-loaded PLGA NPs (with or without double functionalization), a fluorescence signal was observed due to the interaction of PLGA NPs with bacteria. Quantitative analysis shows that non-modified PLGA NPs interacted with 71 ± 3% of the bacterial population (Figure 5B), whereas FA-modified (Figure 5C) and TAT-modified NPs (Figure 5D) showed higher interaction (84 ± 1% and 83.2 ± 0.1%, respectively) (*p* < 0.05). This can be attributed to the presence of the TAT peptide at the NPs’ surface, which increases the membrane permeability and leads to enhanced NPs’ uptake by bacteria [38]. Moreover, since folate receptors are upregulated in *S. aureus* [17], modifying NPs with FA improves their internalization into bacteria [39]. Double-modified FA/TAT PLGA NPs (Figure 5E) exhibited the highest interaction (87 ± 1%) (*p* < 0.5), suggesting a possible additive effect of the two ligands in promoting the interaction with *S. aureus*.

### 2.4. Antibacterial Properties of NPs

**Figure 6:**
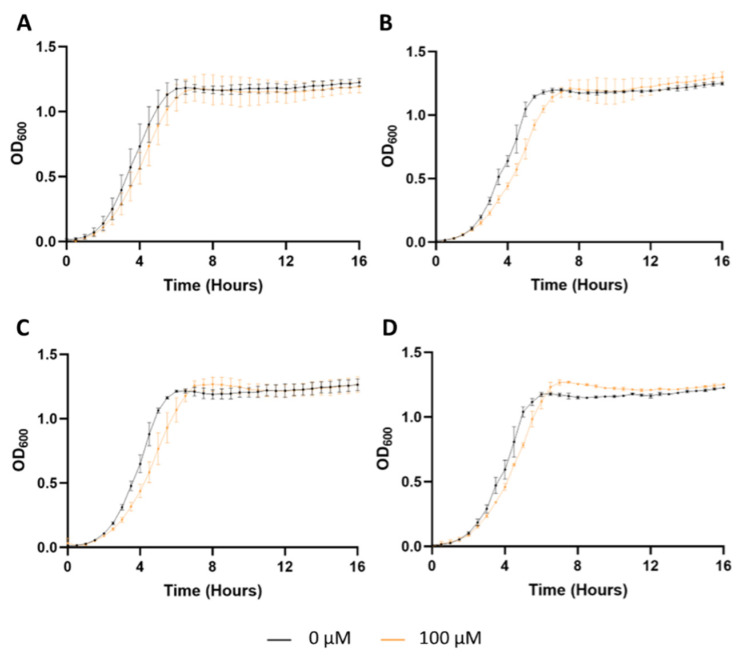
Growth curves of *S. aureus* incubated with (**A**) non-modified, (**B**) FA-modified, (**C**) TAT-modified, or (**D**) FA/TAT-modified PLGA NPs at 0 (black) and 100 µM (orange).

An in vitro experiment was conducted to investigate the antibacterial properties of the produced NPs. *S. aureus* was incubated with increasing concentrations of NPs (0 to 100 µM) at 37 °C for 16 h. OD_600_ values were recorded over time. Figure 6 shows the obtained growth curves for *S. aureus* alone and with non-decorated and FA-, TAT-, and FA/TAT-decorated PLGA NPs.

As illustrated in Figure 6, the bacterial growth curves can be clearly divided into three characteristic phases: lag, exponential, and stationary. During the lag phase, the cells remain metabolically active, synthesizing essential proteins, enzymes, and other biomolecules, but do not undergo division. This preparatory period allows bacteria to adapt to the new environment and optimize their metabolic machinery. Following this, the exponential phase begins, marked by rapid and continuous cell division, leading to a steep increase in population density. Eventually, growth reaches a plateau, known as the stationary phase, in which the rate of new cell formation balances the rate of cell death. This phase reflects limitations in nutrients, accumulation of waste products, and other environmental constraints that collectively stabilize the overall population size. Understanding these phases is critical for interpreting how nanoparticles or other treatments influence bacterial proliferation dynamics over time [40].

Equations (5) and (6) were applied to model the experimental data, allowing the determination of key kinetic parameters for the bacterial growth curves. The calculated parameters include the duration of the lag phase, the half-time of growth, the growth rate constant, and the maximum OD_600_. A comprehensive summary of these values is presented in Table 2, providing a quantitative basis for comparing the effects of different nanoparticle treatments on *S. aureus* proliferation. The models showed excellent goodness-of-fit for all conditions, with R^2^ values above 0.9911, thus confirming the reliability of the fitted curves.

As detailed in Table 2, no significant variations were observed when PLGA NPs were added (compared to the control, *S. aureus* alone) on the kinetic parameters evaluated (*p* > 0.05), including lag time, half-time, growth rate, and maximum OD_600_ values, implying that PLGA NPs per se have no antibacterial properties nor promote bacterial growth regardless of the tested NPs concentration. Similar conclusions were drawn previously by Ucak et al. (2020) [41].

However, a different pattern was observed when incubating *S. aureus* with FA-, TAT-, and FA/TAT-modified PLGA NPs, as displayed in Figure S2B–D, respectively. FA-decorated NPs did not affect the lag phase time (*p* > 0.05) but significantly increased the t_1/2_ in a concentration-dependent manner (*p* < 0.05), from 3.8 ± 0.1 to 4.5 ± 0.2 h, inferring that FA increased the time needed to reach 50% of the maximum OD_600_ value, resulting in a reduction in the growth rate from 1.38 ± 0.04 to 1.06 ± 0.04 h^−1^ (*p* < 0.05). Moreover, a significant increase in OD_600_ values, reflecting an increased cell count, was observed for all the tested concentrations (*p* < 0.05), indicating that FA promotes *S. aureus* division. In fact, FA, a member of the B complex group (B9), is a vitamin of exogenous origin crucial for various cells, including bacteria. It plays a vital role in DNA replication, repair, methylation, and cellular metabolic activities [42].

Likewise, the highest concentration of TAT-modified PLGA NPs (100 µM) led to a significant increase in the t_1/2_ from 3.8 ± 0.2 to 4.6 ± 0.3 h (*p* < 0.05), revealing a slowdown in cell division. Moreover, a rise in the lag time from 2.5 ± 0.1 to 3.0 ± 0.2 h was verified at 100 µM (*p* < 0.05), denoting a delay in the beginning of bacterial division. These findings align with the results reported by Zhu et al. (2009) [43], who demonstrated the strong antibacterial properties of the TAT peptide against a wide range of pathogens, including *S. aureus*. At lower concentrations, TAT-modified PLGA NPs did not affect the growth curve of *S. aureus* (*p* > 0.05) (Figure 6C).

Indeed, as depicted in Table 2, it is evident that the FA/TAT-modified NPs also influenced the growth curve of *S. aureus*, as shown in Figure 6D. Significant differences were observed in all kinetic parameters evaluated (*p* < 0.05) in a concentration-dependent manner. As verified for FA-functionalized NP, FA/TAT-modified NPs at all tested concentrations also slightly increased the maximum OD_600_ value measured. Moreover, a significant increase in the lag time and t_1/2_ was verified for the highest NP concentration, attributed to TAT and FA at the NPs’ surface, respectively.

### 2.5. Biocompatibility

**Figure 7:**
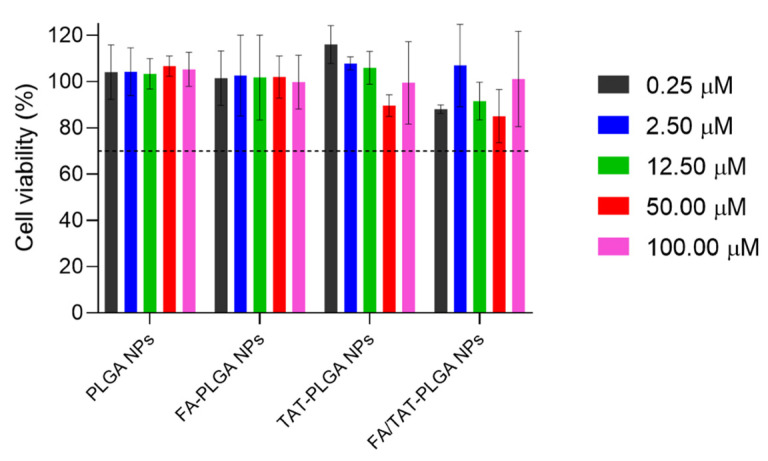
In vitro cell viability assay of non-modified, FA-, TAT-, and FA/TAT-modified PLGA NPs at increasing concentrations. The dashed line indicates the cytotoxicity threshold, representing 70% of cell viability compared to the negative control.

The biocompatibility of nanoformulations is a critical parameter that must be guaranteed to ensure the safety of the prepared NPs towards healthy cells. Therefore, the in vitro biocompatibility of non-decorated and FA-, TAT-, and FA/TAT-decorated PLGA NPs at increasing concentrations (0–100 µM) was investigated using the SRB cellular cytotoxicity assay. Human dermal fibroblast cells (HDFn) were used in this experiment due to the high rate of skin infections caused by *S. aureus* [44]. Figure 7 presents the results of the cell viability assay.

As illustrated in Figure 7, the assessment of cell viability revealed that fibroblast cells maintained levels close to 100% following exposure to PLGA NPs across all tested concentrations, with no statistically significant differences observed (*p* > 0.05). These findings indicate that the NPs themselves do not induce cytotoxic effects within 24 h of treatment, corroborating the well-documented biocompatibility and safety profile of PLGA-based nanocarriers [45,46]. Moreover, the surface functionalization of PLGA NPs with FA, the TAT peptide, or a combination of both ligands did not produce any detectable decrease in cell viability relative to untreated control cells, independent of the NPs concentration employed. This outcome demonstrates that the chemical modifications introduced to achieve targeted delivery do not compromise the inherent cytocompatibility of the PLGA system. Taken together, these results provide strong evidence that the engineered FA/TAT-functionalized NPs are highly biocompatible and suitable for application as drug delivery vehicles, supporting their potential for safe in vivo use without inducing acute cytotoxicity in non-target mammalian cells.

## 3. Materials and Methods

### 3.1. Materials

PLGA Resomer^®^ RG 503 H (lactide/glycolide 50:50, MW 24,000–38,000 Da), FA (MW 441 Da, purity ≥ 97%), Mowiol^®^ 4-88 (polyvinyl alcohol, PVA, MW 31,000 Da), *N*-(3-dimethylaminopropyl)-N′-ethylcarbodiimide hydrochloride (EDC, MW 192 Da, purity ≥ 95%), rhodamine B (MW 479 Da, purity ≥ 95%), sodium chloride (NaCl, MW 58 Da, purity ≥ 99%), sodium hydroxide (NaOH, MW 40 Da, purity > 97%), acetic acid (MW 60 Da, purity ≥ 98%), trichloroacetic acid (TCA, MW 163 Da, purity > 99%), and α-MEM (Minimum Essential Medium—Alpha Modification) were acquired from Sigma-Aldrich (St. Louis, MO, USA). Sulforhodamine B (SRB, MW 581 Da) and tris(hydroxymethyl)aminomethane (Tris base, MW 121 Da, purity ≥ 99%) were purchased from Alfa Aesar (Haverhill, MA, USA). TAT peptide (GRKKRRQRRRPQ, MW 1622, purity ≥ 95%) was obtained from GenScript Biotech (Piscataway, NJ, USA). Tryptic soy broth (TSB) and agar powder were acquired from Millipore (Burlington, MA, USA) and VWR (Radnor, PA, USA), respectively. Fetal bovine serum (FBS), penicillin, and streptomycin were acquired from Gibco (New York, NY, USA). Human dermal fibroblasts (HDFn) were purchased from Institute Corriel (Camden, NJ, USA) and *S. aureus* CECT 976 (*S. aureus*) from the Spanish Type Culture Collection (Valencia, Spain). Uranyl acetate dihydrate (MW 424, purity > 99%) was obtained from Electron Microscopy Sciences (Hatfield, PA, USA). Ultrapure water (Milli-Q Academic, Millipore, Merck KGaA, Burlington, MA, USA) was used to prepare HEPES buffer (pH 7.4, 10 mM), purchased from Sigma-Aldrich (St. Louis, MO, USA) and AppliChem (Darmstadt, Germany), respectively.

### 3.2. Production of PLGA NPs

PLGA NPs were produced by the single emulsion-solvent evaporation method, as previously described [47]. Briefly, 50.0 mg of PLGA was dissolved in 2.0 mL of ethyl acetate. Then, 4.0 mL of PVA 1% (*w*/*v*) in ultrapure water was added dropwise and vortexed for 10 s. The oil-in-water emulsion was subjected to sonication for 60 s (10 s ON, 10 s OFF) in an ice-cold water bath using an ultrasonic processor UP400S (Hielscher, Teltow, Germany) with an amplitude of 70% and ultrasonic frequency of 24 kHz. The emulsion was then magnetically stirred (Multistirrer, VELP Scientifica, Usmate, Italy) for 1 h at room temperature for ethyl acetate complete evaporation. The NPs suspension was stored at 4 °C for 2 h before being collected by a sequence of centrifugation steps with increasing speeds (1 min at 6000 to 14,500 rpm, in intervals of 2000 rpm, followed by cycles of 5, 10, and 15 min at 14,500 rpm) (MiniSpin^®^plus, Eppendorf, Germany). The supernatant was removed, and the pellet containing PLGA NPs was resuspended in ultrapure water at a final concentration of 50 mg/mL by gently vortexing the sample to ensure homogeneity. The NP suspension was stored at 4 °C for further analysis and functionalization.

### 3.3. Functionalization of NPs with FA and TAT

The surface of PLGA NPs was functionalized with FA, TAT, or FA/TAT using the water-soluble carbodiimide coupling reagent EDC [48]. EDC activates PLGA’s carboxyl functional groups to form amine-reactive intermediates that spontaneously react with primary amines of FA and TAT to form amide bonds. A total of 500 molecules of FA, TAT, or both were added per NP. To determine this ratio, the number of PLGA NPs in the suspension was estimated using the following equations:

(1) $$Number\;of\;PLGA\;NPs=\frac{Total\;volume\;of\;NPs({cm}^{3})}{Volume\;of\;a\;single\;NP\;({cm}^{3})}$$

(2) $$Total\;volume\;of\;NPs\left({cm}^{3}\right)=\frac{Mass\;of\;PLGA\;(g)}{Densitity\;of\;PLGA\;(g/{cm}^{3})}$$

(3) $$Volume\;of\;a\;single\;NP\left({cm}^{3}\right)=\frac{4}{3}\;\pi \;r^{3}$$

where r is the radius of PLGA NPs, in cm.

Shortly, EDC in ultrapure water was added to PLGA NPs at a 20× molar excess. The mixture was vortexed for 2 h at room temperature. Free EDC was then removed by centrifugation with increasing speeds (6000 to 14,500 rpm) and time (1–30 min) (MiniSpin^®^plus, Eppendorf, Germany). The resulting pellet containing PLGA NPs was resuspended in ultrapure water. Activated PLGA NPs (50 mg/mL, 250 µL) were then vortexed with FA (in NaOH) (0.5 mg/mL, 2.87 µL), TAT (in ultrapure water) (1 mg/mL, 5.27 µL), or FA + TAT (NaOH: ultrapure water mixture, 1:1 *v*/*v*) at room temperature for 4 h. This corresponds to 500 molecules of FA or TAT per NP. The molar ratios of FA and TAT relative to PLGA were approximately 0.008:1. Under these conditions, the primary amines of FA and TAT selectively react with the activated PLGA carboxyl groups, while the carboxylic groups of FA and TAT remain unreactive, preventing undesired cross-reactions. Non-conjugated FA and TAT, and EDC byproducts were removed by centrifugation with increasing speeds and time. Functionalized PLGA NPs were resuspended in HEPES buffer and stored at 4 °C for further investigation.

### 3.4. Physicochemical Characterization of NPs

#### 3.4.1. Dynamic Light Scattering and Electrophoretic Light Scattering

Dynamic light scattering (DLS) was used to physicochemically evaluate the hydrodynamic diameter and PDI of the NPs, while electrophoretic light scattering (ELS) was used to assess the NPs’ zeta potential. NP suspensions were diluted in HEPES buffer to a final concentration of 0.1 mg/mL. Measurements were conducted at 25 °C using a ZetaSizer Nano ZS (Malvern Instruments, Malvern, UK) equipped with a 633 nm red laser with a 173° backscattering angle. Zeta potential values were obtained employing the Smoluchowski mathematical model (F(ka) value: 1.50). Water’s viscosity (dispersant), refractive index, and dielectric constant were set at 0.8872 cP, 1.330, and 78.5, respectively, while the refractive index and absorption values of PLGA were defined as 1.590 and 0.010, respectively.

#### 3.4.2. Transmission Electron Microscopy

The NPs’ morphology was investigated by TEM using a JEM 1400 electron microscope (JEOL, Tokyo, Japan) at an accelerating voltage of 80 kV. Briefly, 10 µL of the NP suspension (2 mg/mL) was placed on a Formvar/carbon-400 mesh copper grid (Agar Scientific, Rotherham, UK). After 5 min, the sample was blotted from the grid with a filter paper and negatively stained with a 2% (*w*/*v*) uranyl acetate solution (15 µL) in ultrapure water for 45 s, which was centrifuged at 12,000 rpm for 3 min before use. The solution was removed with filter paper and air-dried before morphological analysis. TEM images were acquired at 12,000×, 25,000×, 50,000×, 80,000×, and 100,000× magnification. The NPs’ size distribution histograms were obtained considering the measurement of 150 NPs per sample using the ImageJ software, version 1.54g (National Institutes of Health, Bethesda, MD, USA). The NPs’ PDI was determined according to the following equation (Equation (4)) [49]:

(4) $${PDI=(\frac{\sigma }{D})}^{2}$$

where *σ* is the standard deviation and *D* is the mean NPs’ diameter.

#### 3.4.3. Fourier-Transform Infrared Spectroscopy

FTIR was used to confirm the conjugation of FA and TAT peptide to PLGA NPs, as previously detailed [50]. Briefly, 3 μL of the FA-, TAT-, and FA/TAT-functionalized PLGA NPs were placed on the surface of the FTIR crystal, and the water was removed using a nitrogen stream. FTIR spectra were then recorded through 64 scans in the absorbance mode from 375 to 4000 cm^−1^ at 2 cm^−1^ resolution intervals using an ALPHA-P spectrophotometer (Bruker Corporation, Billerica, MA, USA). The FTIR spectra of FA, TAT, and PLGA NPs were also collected as controls. In addition, to distinguish chemical conjugation from simple physical mixing, FTIR spectra of physical mixtures of PLGA NPs with FA, TAT, or FA/TAT were also recorded under the same experimental conditions. The background spectrum was also recorded and subtracted from all spectra.

### 3.5. Physicochemical Stability of NPs

The distinct formulations’ colloidal stability, including non-functionalized and FA, TAT, and FA/TAT-functionalized PLGA NPs, was investigated over 4 months while stored at 4 °C. For that, the physicochemical properties of the NPs, including hydrodynamic diameter, PDI, and zeta potential, were evaluated as detailed in Section 3.4.1.

### 3.6. Bacterial Targeting Ability of NPs

The efficacy of the double-functionalization to increase the targeting ability of PLGA NPs towards *S. aureus* was investigated by fluorescence-based flow cytometer analysis. In brief, *S. aureus* (CECT 976 from the Spanish Type Culture Collection, Spain) was cultured in a TSB medium overnight at 37 °C (INCU-Line^®^, VWR, Radnor, PA, USA). The inoculum was then diluted to OD_600_ = 0.3. Five aliquots of the diluted bacterial suspension (1.0 mL) were centrifuged for 15 min at 14,000 rpm and at room temperature (Centrifuge 5148, Eppendorf, Germany). The medium was removed, and bacteria were resuspended with 150 µL of rhodamine B-loaded non-functionalized and FA, TAT, and FA/TAT-functionalized PLGA NPs (10 mg/mL) in HEPES buffer. Rhodamine B-loaded NPs were produced by the double emulsion-solvent evaporation method. The samples were incubated at 37 °C for 1 h under continuous shaking at 160 rpm (IKA^®^KS 130 basic, Staufen, Germany). Then, bacteria-bonded NPs were separated from non-bonded NPs by filtering the samples through a Nuclepore™ track-etched polycarbonate membrane (Cytiva, Marlborough, MA, USA) with pores of 400 nm. Samples’ fluorescence intensities were quantified using the CytoFLEX Flow Cytometer (Beckman Coulter, Brea, CA, USA) equipped with a 488 nm excitation laser and two fluorescence channels, 585/42 BP and 690/50 BP. A minimum of 10,000 events per sample was collected. Data were analyzed using the CytExpert 2.4.0.28 software (Beckman Coulter, Brea, CA, USA).

### 3.7. Antibacterial Properties of NPs

#### 3.7.1. Growth Curve of *S. aureus*

The antibacterial activity of the FA/TAT-functionalized PLGA NPs was investigated by assessing the growth curves of *S. aureus* in the presence or absence of NPs. Briefly, *S. aureus* was cultured in TSB medium overnight at 37 °C under continuous agitation at 160 rpm (IKA^®^KS 130 basic, Germany). 100 µL of the bacterial suspension was added to 10 mL of TSB medium. 200 µL of the diluted bacterial suspension and 20 µL of PLGA NPs in HEPES buffer were mixed in a 96-well plate (polystyrene, non-pyrogenic, tissue culture treated, flat bottom, Orange Scientific) to a final NP concentration of 0 to 100 µM. The microplate was incubated at 37 °C for 16 h, and OD_600_ values were recorded every 30 min using a FLUOstar^®^ Omega (version 5.70.101) multi-mode microplate reader (BMG LABTECH, Ortenberg, Germany). Before each measurement, the microplate was shaken for 100 s at 200 rpm. The growth curves were plotted accordingly as OD_600_ values vs. time (hours). The growth curves of *S. aureus* in the presence or absence of FA- and TAT-functionalized NPs were also recorded for comparison. The OD_600_ of the controls (samples without *S. aureus*) was subtracted from the respective samples.

#### 3.7.2. Kinetic Model of *S. aureus* Growth

The kinetic parameters of *S. aureus* growth curves were assessed by fitting Equation (5) to the obtained OD_600_ values over time (t):

(5) $${OD}_{600}(t)={OD}_{600}(0)+\frac{{OD}_{600}(16)}{1+e^{\left[-k\left(t-t_{1/2}\right)\right]}}$$

where OD_600_ (0) and OD_600_ (16) correspond to the OD_600_ values at the beginning and end of the experiment, respectively. The growth rate constant is denoted by *k*, and the time needed to reach half of OD_600_ (16) is represented by *t*_1/2_.

The lag time of *S. aureus* growth curves (*t_lag_*) was calculated using Equation (6):

(6) $$t_{lag}=t_{1/2}-\frac{2}{k}$$

### 3.8. Biocompatibility of NPs

#### 3.8.1. Cell Culture

Human dermal fibroblast cells (HDFn) were used to assess the NPs’ biocompatibility. These cells were cultured in α-MEM cell culture medium with 10% (*v*/*v*) FBS and 1% (*v*/*v*) penicillin-streptomycin (100 U/mL penicillin, 100 mg/mL streptomycin). The cells were kept in a 5% CO_2_ atmosphere at 37 °C.

#### 3.8.2. Cytotoxicity Evaluation

The biocompatibility of non-functionalized and FA, TAT, and FA/TAT-functionalized PLGA NPs at increasing concentrations (0–100 µM) was evaluated using the SRB cellular cytotoxicity assay. Briefly, 2000 cells/well (10^4^ cells/mL) were seeded in a 96-well plate (polystyrene, non-pyrogenic, tissue culture treated, flat bottom, Orange Scientific) and left to adhere for 24 h at 37 °C. After this period, the medium was replaced by fresh medium, and PLGA NPs in HEPES buffer were added. The plate was incubated for 24 h at 37 °C. After the treatment, the medium was removed, and the cells were fixed with 100 μL of a TCA solution (10% *w*/*v*) at 4 °C for 1 h. The plate was washed with ultrapure water and dried at 37 °C (VENTI-Line^®^ Prime, VWR, Radnor, PA, USA) for 10 min. Cells were stained with 50 μL/well of a SRB solution (0.4% *w*/*v*) in acetic acid (1% *v*/*v*) for 20 min at room temperature. The plate was washed twice with 1% acetic acid solution (400 μL/well) to remove unbound SRB and dried at 37 °C for 10 min. The protein-bound SRB was solubilized by adding 100 μL of Tris-base buffer to the wells. The samples’ UV-Vis absorbances at 560 nm were recorded utilizing a Synergy™ 2 Multi-Mode Microplate Reader (BioTek Instruments, Winooski, VT, USA). Data were treated, and cell survival was determined as a percentage (% = Absorbance of treated cells/Absorbance of untreated cells × 100). The percentage of cell survival as a function of PLGA NPs’ concentration was plotted.

### 3.9. Statistical Analysis

All experiments were conducted at least in triplicate, and the results are expressed as mean  ±  standard deviation. Student *t*-tests with a 95% confidence interval were employed to assess if there were significant differences between groups. *p*-values below 0.05 were used to identify significant differences.

## 4. Conclusions

Concluding, this work introduces and evaluates a dual-modified PLGA-based nanosystem designed for the treatment of *S. aureus* infections. The NPs were consistently below 180 nm, which is an appropriate size for drug delivery purposes. FTIR analysis clearly confirmed the successful surface modification with FA and TAT peptide, while stability testing showed that the formulation could be stored without losing its properties.

More importantly, the addition of FA and TAT increased the interaction between the NPs and *S. aureus*, proving that the chosen ligands effectively improved targeting. The particles also showed good compatibility with human dermal fibroblast cells, suggesting they are safe enough to move toward in vivo evaluation. Interestingly, besides serving as carriers, the FA/TAT-modified NPs displayed a slight but measurable antibacterial effect on their own by influencing the bacterial growth curve.

While the current study focuses on the development and thorough physicochemical characterization of a versatile dual-functionalized PLGA NP platform without encapsulating a specific antibiotic, this represents a limitation regarding direct therapeutic evaluation. The study was designed to establish a generic carrier with well-defined size, stability, surface modification, and targeting properties, suitable as a modular system for a variety of antimicrobial agents or other bioactive compounds. Future studies can build on these findings by loading the NPs with known antibiotics, enabling investigation of targeted delivery, controlled release, and potential synergistic effects with the FA/TAT functionalization. While hydrophobic molecules, such as rifampicin, can be incorporated into the PLGA matrix via single-emulsion (oil-in-water) solvent evaporation, hydrophilic drugs, such as gentamicin, can be encapsulated using a double-emulsion (water-in-oil-in-water) method to ensure efficient loading. Such studies will provide insights into the therapeutic potential of this platform, including efficacy against resistant strains and safety under physiologically relevant conditions.

Bringing these findings together, these outcomes point to the FA/TAT-functionalized nanocarriers as a promising platform for selective antimicrobial delivery. By combining stability, safety, and improved bacterial binding, this nanosystem could offer a useful strategy for tackling *S. aureus* infections and may also contribute to efforts aimed at slowing down the growing problem of antibiotic resistance.

## Figures and Tables

**Table 1:** Physicochemical properties of non-decorated and FA-, TAT-, and FA/TAT-decorated PLGA NPs in HEPES buffer (pH 7.4, 10 mM) (n = 4) determined by dynamic light scattering (DLS) and electrophoretic light scattering (ELS). * Indicates a statistical difference compared to non-decorated PLGA NPs (*p* < 0.05).

Formulation	Mean Diameter (nm)	PDI	Zeta Potential (mV)
PLGA NPs	159 ± 12	0.06 ± 0.02	−3.6 ± 1.7
FA-decorated PLGA NPs	174 ± 8 *	0.07 ± 0.02	−2.8 ± 1.1
TAT-decorated PLGA NPs	188 ± 8 *	0.05 ± 0.02	−2.6 ± 1.1
FA/TAT-decorated PLGA NPs	174 ± 4 *	0.08 ± 0.02	−2.5 ± 0.1

**Table 2:** Kinetic parameters of *S. aureus* growth curves in the absence and presence of increasing concentrations of non-modified, FA-, TAT-, and FA/TAT-modified PLGA NPs. Values were estimated using Equations (5) and (6). * *p* < 0.05 indicates a statistical difference compared to the respective control (untreated *S. aureus*).

Formulation	NP Concentration (µM)	t_lag_ (h)	t_1/2_ (h)	k (h^−1^)	Max. OD_600_	R^2^
PLGA NPs	0	2.1 ± 0.3	3.6 ± 0.4	1.4 ± 0.1	1.19 ± 0.03	0.9981 ± 0.0003
	25	2.4 ± 0.3	3.7 ± 0.4	1.5 ± 0.1	1.21 ± 0.04	0.9979 ± 0.0006
	50	2.4 ± 0.3	3.9 ± 0.4	1.4 ± 0.1	1.21 ± 0.03	0.9981 ± 0.0009
	100	2.4 ± 0.4	4.0 ± 0.5	1.3 ± 0.1	1.17 ± 0.08	0.9959 ± 0.0034
FA-decorated PLGA NPs	0	2.4 ± 0.1	3.8 ± 0.1	1.38 ± 0.04	1.19 ± 0.01	0.9910 ± 0.0057
	25	2.6 ± 0.1	4.0 ± 0.2	1.4 ± 0.1	1.23 ± 0.01 *	0.9930 ± 0.0070
	50	2.6 ± 0.2	4.3 ± 0.1 *	1.18 ± 0.04 *	1.27 ± 0.02 *	0.9942 ± 0.0048
	100	2.7 ± 0.3	4.5 ± 0.2 *	1.06 ± 0.04 *	1.24 ± 0.03 *	0.9844 ± 0.0091
TAT-decorated PLGA NPs	0	2.5 ± 0.1	3.8 ± 0.2	1.5 ± 0.2	1.21 ± 0.05	0.9974 ± 0.0001
	25	2.7 ± 0.1	4.0 ± 0.2	1.4 ± 0.1	1.19 ± 0.04	0.9979 ± 0.0010
	50	2.8 ± 0.2	4.3 ± 0.3	1.3 ± 0.1	1.22 ± 0.05	0.9974 ± 0.0006
	100	3.0 ± 0.2 *	4.6 ± 0.3 *	1.3 ± 0.1	1.21 ± 0.06	0.9961 ± 0.0019
FA/TAT-decorated PLGA NPs	0	2.5 ± 0.2	3.9 ± 0.2	1.47 ± 0.03	1.16 ± 0.01	0.9958 ± 0.0011
	25	2.6 ± 0.1	4.0 ± 0.1	1.4 ± 0.1	1.20 ± 0.01 *	0.9977 ± 0.0005
	50	2.8 ± 0.1	4.2 ± 0.1	1.37 ± 0.01 *	1.19 ± 0.01 *	0.9977 ± 0.0005
	100	2.88 ± 0.04 *	4.4 ± 0.1 *	1.3 ± 0.1 *	1.21 ± 0.01 *	0.9953 ± 0.0019

## Data Availability

The raw data supporting the conclusions of this article will be made available by the authors on request.

## Supplementary Materials

The following supporting information can be downloaded at: https://www.mdpi.com/article/10.3390/ijms262110666/s1.

## Abbreviations

The following abbreviations are used in this manuscript:

DDS	Drug delivery system
DLS	Dynamic light scattering
EDC	*N*-(3-dimethylaminopropyl)-N′-ethylcarbodiimide hydrochloride
ELS	Electrophoretic light scattering
FBS	Fetal bovine serum
FA	Folic acid
FTIR	Fourier transform infrared spectroscopy
NPs	Nanoparticles
PDI	Polydispersity index
PLGA	Poly(lactic-co-glycolic acid)
PVA	Polyvinyl alcohol
TEM	Transmission electron microscopy
TSB	Tryptic soy broth
