# Supplementary material for: PLGA Nanoparticles Double-Decorated with a TAT Peptide and Folic Acid to Target Staphylococcus aureus
Source: Int J Mol Sci. 2025 Nov 1;26(21):10666. doi: 10.3390/ijms262110666 (PMC12608785; doi:10.3390/ijms262110666)
Supplement: Supplementary file 1 [file ijms-26-10666-s001.zip › ijms-3937177-supplementary.pdf]

SUPPLEMENTARY INFORMATION

PLGA Nanoparticles Double-Decorated with a TAT Peptide and Folic Acid to Target *Staphylococcus aureus*

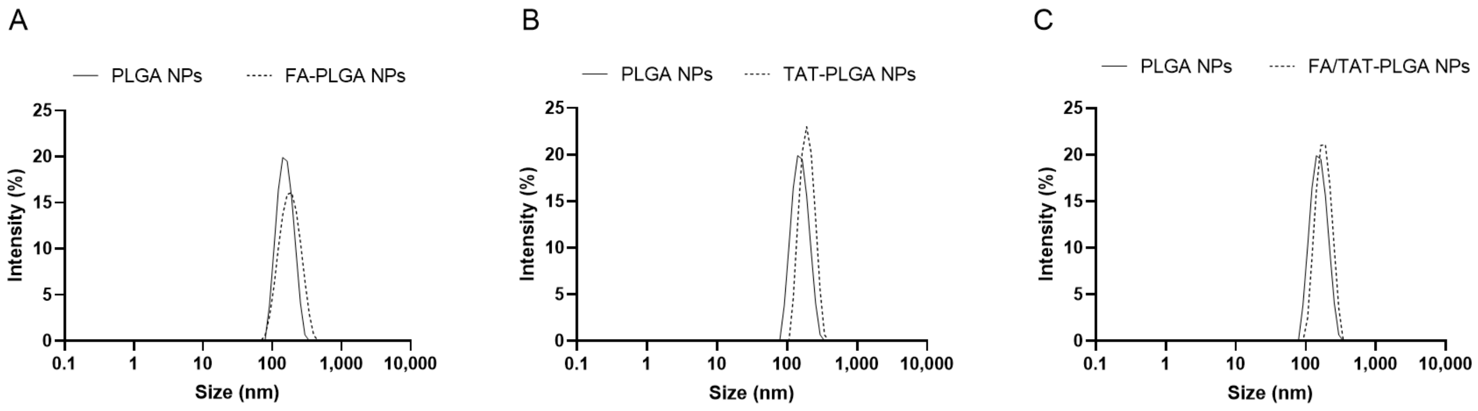

Figure S1. Size distribution by intensity curves of PLGA NPs obtained by DLS. Each panel includes the size distribution of non-modified PLGA NPs and those modified with **A)** FA-modified, **B)** TAT-modified, or **C)** FA/TAT-modified PLGA NPs.

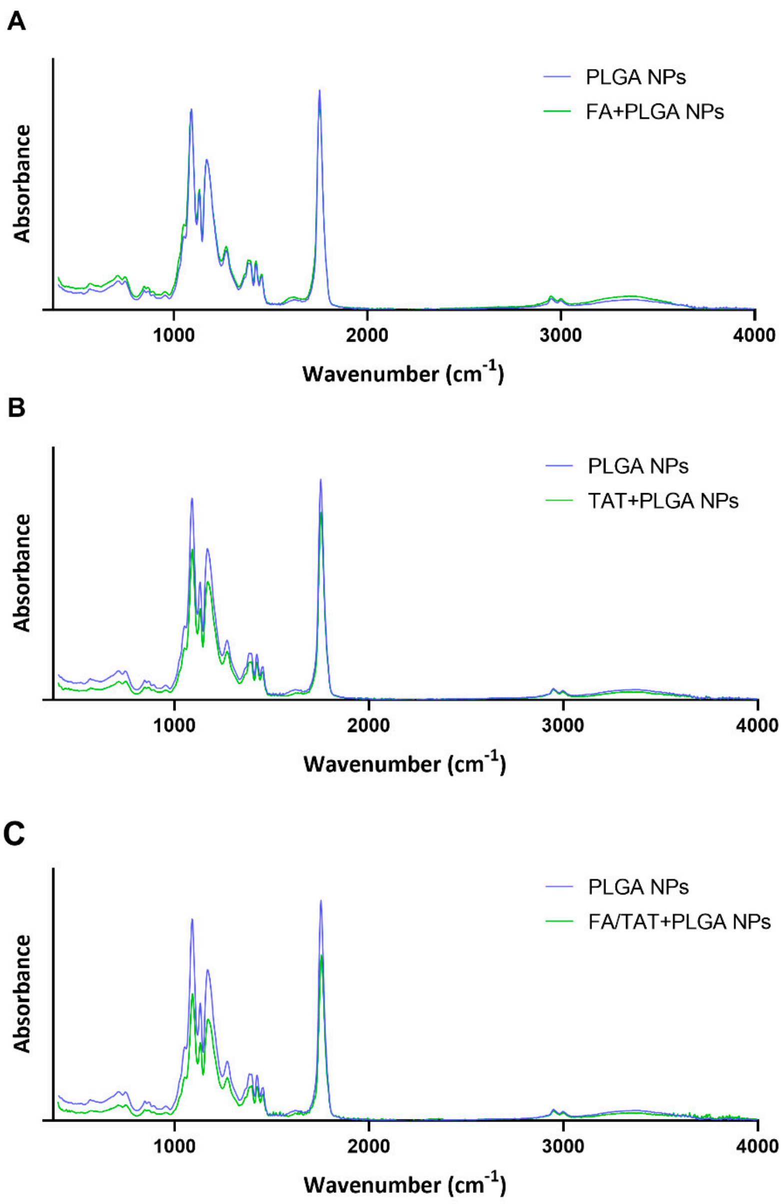

Figure S2. FTIR spectra of PLGA NPs mixed with (**A**) FA, (**B**) TAT, and (**C**) FA+TAT.

Table S1. Physicochemical properties of non-loaded and rhodamine B-loaded PLGA NPs in HEPES buffer (pH 7.4, 10 mM) (n=3) determined by DLS and ELS.

Formulation	Mean diameter (nm)	PDI	Zeta potential (mV)
**Unloaded PLGA NPs**	159 ± 12	0.06 ± 0.02	-3.6 ± 1.7
**Rhodamine B-loaded PLGA NPs**	145 ± 3	0.08 ± 0.01	-2.1 ± 0.5
